# Prognostic Factors of Low-Grade Gliomas in Adults

**DOI:** 10.3390/curroncol29100576

**Published:** 2022-09-30

**Authors:** Mariana Deacu, Steliana Popescu, Any Docu Axelerad, Theodor Sebastian Topliceanu, Mariana Aschie, Madalina Bosoteanu, Georgeta Camelia Cozaru, Ana Maria Cretu, Raluca Ioana Voda, Cristian Ionut Orasanu

**Affiliations:** 1 Clinical Service of Pathology, Departments of Pathology, Sfantul Apostol Andrei Emergency County Hospital, 900591 Constanta, Romania; 2 Faculty of Medicine, “Ovidius” University of Constanta, 900470 Constanta, Romania; 3 Department of Radiology, Sfantul Apostol Andrei Emergency County Hospital, 900591 Constanta, Romania; 4 Department of Neurology, Sfantul Apostol Andrei Emergency County Hospital, 900591 Constanta, Romania; 5 Center for Research and Development of the Morphological and Genetic Studyies of Malignant Pathology (CEDMOG), Ovidius University of Constanta, 900591 Constanta, Romania; 6 Academy of Medical Sciences of Romania, 030167 Bucharest, Romania; 7 Clinical Service of Pathology, Departments of Genetics, Sfantul Apostol Andrei Emergency County Hospital, 900591 Constanta, Romania

**Keywords:** CDKN2A, IDH1, imaging, Ki-67, low-grade glioma

## Abstract

Adult low-grade gliomas are a rare and aggressive pathology of the central nervous system. Some of their characteristics contribute to the patient’s life expectancy and to their management. This study aimed to characterize and identify the main prognostic factors of low-grade gliomas. The six-year retrospective study statistically analyzed the demographic, imaging, and morphogenetic characteristics of the patient group through appropriate parameters. Immunohistochemical tests were performed: IDH1, Ki-67, p53, and Nestin, as well as FISH tests on the CDKN2A gene and 1p/19q codeletion. The pathology was prevalent in females, with patients having an average age of 56.31 years. The average tumor volume was 41.61 cm^3^, producing a midline shift with an average of 7.5 mm. Its displacement had a negative impact on survival. The presence of a residual tumor resulted in decreased survival and is an independent risk factor for mortality. Positivity for p53 identified a low survival rate. CDKN2A mutations were an independent risk factor for mortality. We identified that a negative prognosis is influenced by the association of epilepsy with headache, tumor volume, and immunoreactivity to IDH1 and p53. Independent factors associated with mortality were midline shift, presence of tumor residue, and CDKN2A gene deletions and amplifications.

## 1. Introduction

The low-grade gliomas are grouped by the World Health Organization (WHO) as primary tumors of the central nervous system with histopathological grade 1 and 2 [1]. This category includes both circumscribed and diffuse entities: pilocytic astrocytoma, pleomorphic xantoastrocytoma, oligodendroglioma grade 2, and astrocytoma grade 2 (diffuse astrocytoma) [2,3]. Their main characteristic is represented by a low proliferative index [1]. They have an increased incidence in males between 35–44 years [3]. Unlike the pediatric population, the adult population is less often affected [2].

Life expectancy is relatively good, with an average survival rate of 5.6 to 13.3 years [1]. A number of molecular and constitutional characteristics contribute to this rate: topography, topographic effects, tumor histology, patient age, type of treatment, tumor volume, and patient performance status [1,2,3,4,5]. Another aspect not to be neglected is the potential for malignant transformation of these tumors, which can vary between 25 and 72% of cases [6]. All these aspects must be taken into account for good case management.

The unpredictable behavior of the tumor makes it difficult to assess risk factors. However, to date, studies have only shown the following potential indicators of the promotion of malignancy: age of patient, presence of seizures, topography and tumor volume, molecular status of IDH and P53 genes, and treatment method [6,7,8].

Unlike high-grade gliomas, where studies have clearly highlighted the main negative prognostic factors, the situation of low-grade gliomas has not been fully clarified. Therefore, this study aimed at a clinical-imaging and morphogenetic characterization of cases of low-grade gliomas in adults, as well as the identification of the main prognostic factors through immunohistochemistry and cytogenetic ancillary studies.

## 2. Materials and Methods

We conducted a retrospective study for a period of six years (2012–2017) of patients diagnosed with a central nervous system tumor hospitalized at the Constanta County Emergency Clinical Hospital, Dobrogea region. The data were extracted from the hospital’s archive and electronic databases. The inclusion criteria consisted of adult patients (over 18 years), and histopathological grades 1 and 2. The exclusion criteria consisted of recurrences and necroptic diagnosed cases.

The clinical information (gender, age, clinical presentation) and evolutionary information (relapses, death, treatment) of the patients came from the hospitalization form. These data were evaluated by the neurologist and the attending physician.

Preoperative imaging examinations (magnetic resonance imaging) were aimed at the topography, size, volume, peritumoral edema, and midline shift. Intraoperative gross ablation of the lesion was performed under magnification. The quality of the resection was assessed by magnetic resonance imaging (MRI) performed postoperatively, which measured the remaining residual volume. The examinations were performed at the hospital and were evaluated by a neurologist and radiologist.

Tumorectomy specimens were described macroscopically and prepared according to international protocols, up to the stage of microscopic slides in Hematoxylin–Eosin (HE) staining within the Clinical Anatomic Pathology Service of Constanta. Histopathological evaluation was performed by two pathologists, according to the latest WHO criteria (2021 edition) for the classification of tumors of the central nervous system.

The immunohistochemical examinations were performed at the Center for Research and Development of The Morphological and Genetic Studies of Malignant Pathology (CEDMOG), the evaluation being performed by two different pathologists. Formalin-fixed paraffin-embedded were sectioned at 4 µm and prepared according to the working protocol provided by the manufacturer, Master Diagnostica (Sevilla, Spain), using positive control slides at each execution.

Immunohistochemical tests used the markers IDH1 R132H (H09, ready-to-use, HIER-DAB method), Nestin (10C2, ready-to-use, HIER-DAB method), Ki-67 (SP6, ready-to-use, HIER-DAB method), and p53 (SP5, dilution 1:50, HIER-DAB method). The counter-staining was performed with Hematoxylin–Eosin. Cytoplasmic markers (IDH1 R132H and Nestin) were examined throughout the section, assessing the reactivity and intensity of the reaction. For the nuclear markers (Ki-67 and p53) the reference index was calculated as the percentage of positive nuclei after counting the 10 HPF of at least 1000 nuclei. In the case of the p53 marker, an expression of more than 10% was considered positive.

**Figure 1:**
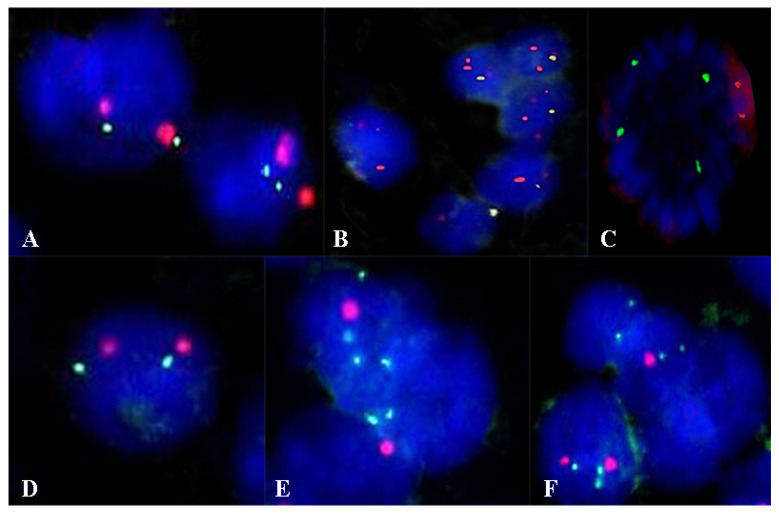
Aspects of the FISH examination in the evaluation of the CDKN2A gene (**A**–**C**) and the 1p/19q codeletion (**D**–**F**). (**A**) Normal status of the CDKN2A gene. (**B**) Deletion at the level of the CDKN2A gene. (**C**) Amplification at the level of the CDKN2A gene. (**D**) Normal status. (**E**) Deletion at the 1p36 locus. (**F**) Deletion at the 19p13 locus. Magnification ×1000.

For the evaluation of fluorescent in situ hybridization (FISH), sections were made from formalin-fixed paraffin-embedded samples sectioned at 3 µm. The tissue slides followed successive pre-treatment, denaturation, hybridization, and post-hybridization stages, according to the protocol developed by the manufacturer. Cytogenetic evaluation used ZytoLight SPEC CDKN2A/CEN 9 Dual Color Probe and ZytoLight Glioma 1p/19q Probe Set probes (Bremerhaven, Germany). Fluorescent signals of the preparations were quantified in 100 tumor nuclei using a fluorescence microscope, Zeiss Axio Imager 2 (Zeiss Gmbh, Germany). In cells without abnormalities, two green and two orange signals were seen. For the CDKN2A gene, in the case of deletion, fewer green signals were observed, and in the case of amplification, more green signals were observed. For Glioma 1p/19q in the case of deletion, less orange signals were observed (Figure 1).

Statistical data analysis was performed in SPSS Statistics Version 26 (IBM Corporation, New York, NY, USA). Central tendency and variability indicators were used. Univariate data analysis was performed using a Chi square test, with Fisher’s exact test for categorical data and *T*-Test, ANOVA, Mann–Whitney U Test, and Kruskal–Wallis H tests for continuous variables, as appropriate. To measure the association of data, we used the Pearson correlation coefficient. Survival estimates were made for a period of 5 years and were calculated using the Kaplan–Meier method and survival differences between groups were analyzed by applying a log-rank test. Hazard ratios (HR) were estimated using Cox regression analysis. Results were considered significant at a *p*-value of <0.05.

All patients signed an informed consent at the time of admission. An ethics opinion was obtained from the local ethics commission (Ethics Commission of the Constanta County Emergency Hospital).

## 3. Results

### 3.1. Demographic and Clinical Characteristics

**Figure 2:**
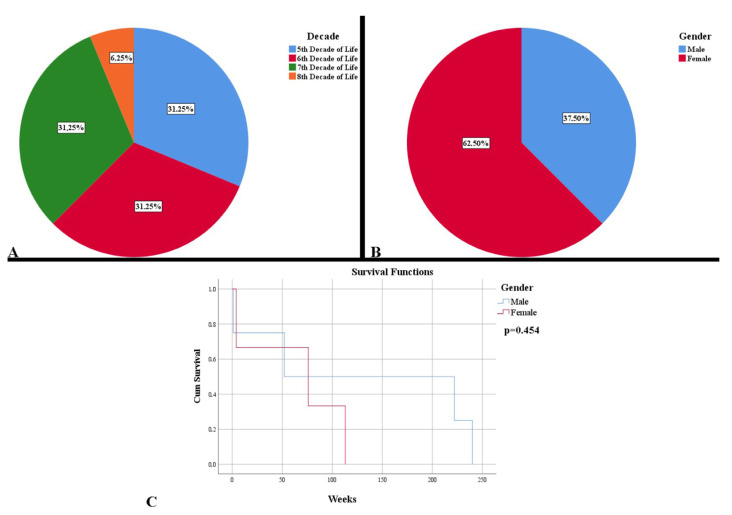
Demographic and clinical characteristics of low-grade gliomas. (**A**) Distribution of cases by decades of age. (**B**) Distribution of cases by gender. (**C**). Kaplan Meier survival graphic that shows a lower survival rate for females.

For the period of six years, we identified 16 cases of low-grade gliomas. The mean age at diagnosis was 56.31 years (41–76), with a uniform distribution in the 5–7 decades of life, with a prevalence in females (Figure 2A,B). There was a lower survival rate for females, of 64.33 weeks, compared to males (128.75 weeks), without any statistical significance (Figure 2C). Age at diagnosis and gender were not an indicator of mortality (HR = 0.99, *p* = 0.758, respectively HR = 0.50, *p* = 0.454).

The symptoms had begun, in most cases, one month (62.50%) before the presentation. The main three manifestations that led to a specialized examination were epileptic seizures (68.75%), headache (50%), and motor deficits (37.50%). There was a statistically significant correlation between the onset of symptoms and gender, noting that the symptoms began in a shorter period before hospitalization (first 3 weeks) in the case of males (*p* = 0.029). In addition, the main association between the clinical picture and gender was represented by headache, females reported headaches more often than men (*p* = 0.041).

In terms of survival, it was observed that the presence of headache was associated with a lower life expectancy (94.5 weeks vs. 103.8 weeks), but without statistical significance (*p* = 0.890). Instead, a predictor of survival was absence of epilepsy (*p* = 0.048). Its presence determined a survival of only 27 weeks vs. 156.75 weeks in the case of those who did not develop seizures.

After the final diagnosis, 87.50% of the patients underwent radio-chemotherapeutic treatment (60 Gy fractionated 5 days out of 7, 2 Gy per session in combination with Temodal–Temozolamide–75 mg/m^2^ for 42 days, followed by monotherapy 150 mg/m^2^ for 5 days). The benefit of this treatment was also reflected in the survival rate, the patients who followed it having an increased average survival rate (*p* = 0.008).

### 3.2. Imaging Features

The imaging examination identified tumor lesions with benign characteristics in 50% of cases. From a topographical point of view, 93.75% of cases were supratentorial, with only one case being identified in the cerebellum. Of these, the left hemisphere was more frequently affected (53.33%). The lesions were identified in 56.25% of cases in eloquent areas in the lobes or border between the lobes, such as the frontal, fronto-parietal, or parietal.

Imaging features captured lesions of varying sizes, most with a maximum diameter of 25–50 mm (56.25%), with an average of 43 mm (15–64 mm). The average volume occupied by tumors was 41.61 cm^3^. Both the maximum tumor diameter and the volume were directly proportional to the presence of headache (*p* = 0.004, respectively *p* = 0.021). A paradoxical element consisted in the increased survival of the patients who presented a larger maximum diameter of lesion (*p* = 0.024). Due to their size, intraparenchymal lesions produced edema of the surrounding tissue in 93.75% of cases, managing to move the midline in the opposite direction of the lesion by an average of 7.5 mm.

**Figure 3:**
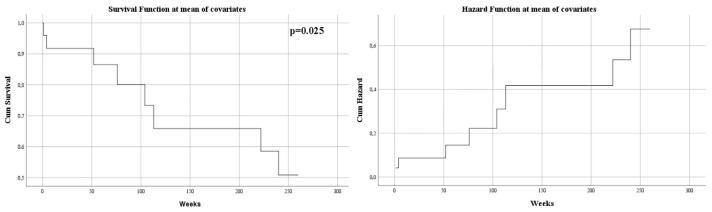
Univariate Cox regression analysis that expresses the risk of death in midline shift.

The mass effect of the tumor, manifested by midline shift, had a negative impact on survival (*p* = 0.028). Its displacement was also an independent risk factor associated with mortality (HR = 1.15, *p* = 0.025) (Figure 3).

**Figure 4:**
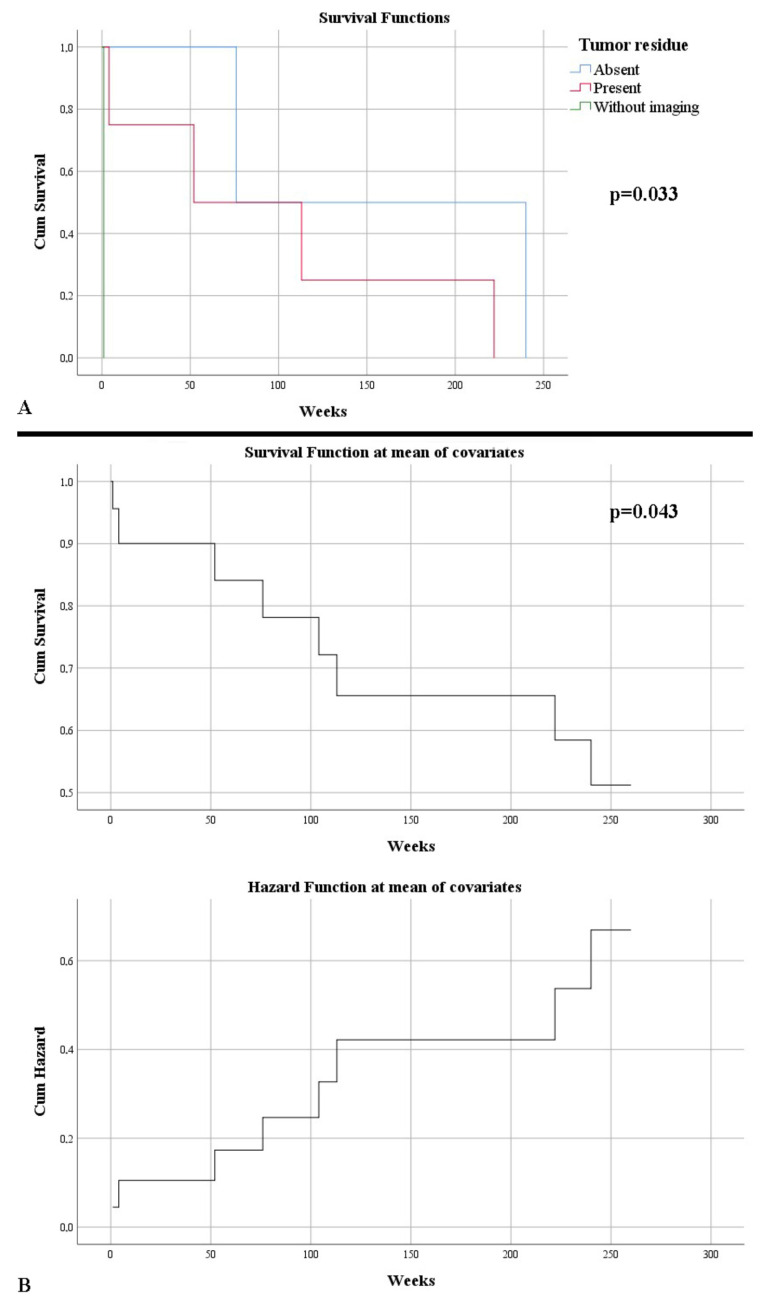
(**A**) Kaplan–Meier survival graphic that shows a lower survival rate in the presence of tumor residue. (**B**) Univariate Cox regression analysis that expresses the risk of death in the presence of a tumor residue.

Evolutionary follow-up of patients revealed a gross total resection as visually proven in only 43.75% of cases. One case did not benefit from a control imaging examination, dying 24 h postoperatively. Thus, a total resectability rate of 92.45% was noted. The residual tumor volume in cases where it was present had an average of 7.82 cm^3^ (0.11–24.84 cm^3^). The residual volume showed a strong statistically significant correlation with the tumor volume, thus large lesions also showed increased residual volumes (*p* < 0.001). An increased residual tumor volume was associated with a lower survival rate (*p* = 0.049), while an increased percentage of remaining tumor was associated with decreased survival (*p* = 0.001). After five years, 50% of the patients survived. Death was on average after 101.14 weeks. As expected, survival was higher in the absence of tumor residue, after complete excision: 158 weeks vs. 97.75 weeks (*p* = 0.033) (Figure 4A). Moreover, this outlines an element of the patient’s independent survival factor (HR = 5.47, *p* = 0.043) (Figure 4B).

### 3.3. Morpho-Genetic Characterization

Histopathological examination identified microscopic features suggestive of four neoplastic entities: pleomorphic xantoastrocytoma (6.25%), oligodendrogliomas (12.50%), pilocytic astrocytoma (25%), and astrocytomas (56.25%).

**Figure 5:**
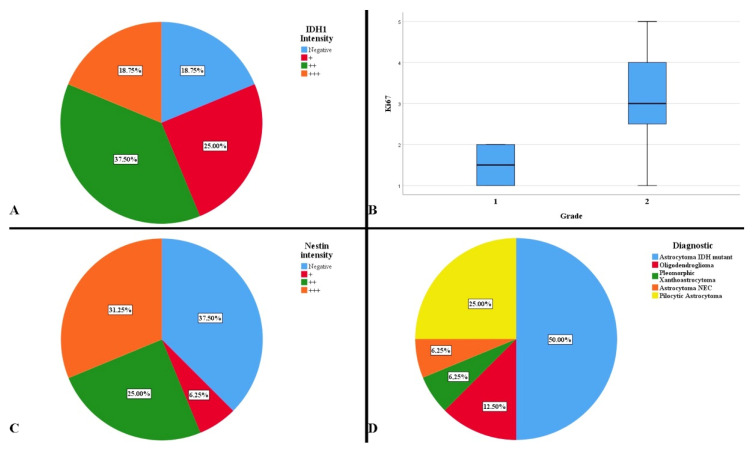
(**A**) Intensity of IDH1 immunoreaction. (**B**) Average distribution of Ki-67 proliferation index by histopathological degrees. (**C**) Intensity of Nestin immunoreaction. (**D**) Complete diagnosis of the studied cases.

In order to establish the exact final diagnosis, additional tests were performed: IDH1 R132H, Ki-67, Nestin, p53, CDKN2A, and 1p/19q codeletion. Two cases presented both IDH1 mutation and the presence of the 1p/19q codeletion; the same cases with a histopathological appearance suggestive of an oligodendroglial tumor. A total of 81.25% of cases were positive for the IDH1 gene mutation, with variations in the reaction intensity (Figure 5A). A statistically significant difference was observed between the degree of intensity of the IDH1 reaction and the midline shift, so that the stronger the reaction, the higher the degree of displacement (*p* = 0.042). This aspect was also noticed in the low survival of the patients, but without a statistically significant significance (*p* = 0.051). The presence of a gradually increasing immunointensity was associated with a low rate of resectability (*p* = 0.009). This intensity did not represent a risk factor of survival (HR = 2.30, *p* = 0.113).

The tumor proliferation index was low, ranging from 1% to 5%, with an average of 2.81% (Figure 5B). A difference was observed in terms of the dominant symptom, headache and proliferation index (*p* = 0.014). The absence of headache correlated with an increased proliferation rate. There was a difference between the immunoreactivity of the IDH1 reaction and the proliferation index, Ki-67 (*p* = 0.017). The weak and strong positive reactions to IDH1 were correlated with an increased proliferation index. Proliferation index was not an independent factor in predicting mortality (HR = 1.344, *p* = 0.291)

The degree of cell differentiation and tumor vascularization were evaluated using the Nestin marker, observing immunoreactivity in 62.50% of cases (Figure 5C). A statistically significant difference was observed between the intensity of the Nestin reaction and gender (*p* = 0.009). A gradual, intense positive reaction mainly occurred in males, while a gradually absent reaction occurred in females. It was observed that moderate and strong intensities were correlated with a higher survival rate than in the cases in which the reaction was weakly positive and absent (*p* = 0.038). However, Nestin was not an independent hazard factor (HR = 0.847, *p* = 0.546).

Immunoreactivity for p53 was observed in 75% of cases, especially grade 2 gliomas. A difference was observed between the reaction to p53 and the histopathological grade, grade 2 gliomas were predominantly positive, as opposed to grade 1 (*p* = 0.027). In addition, p53-positive gliomas produced a more pronounced midline shift (*p* = 0.004). Positivity was also associated with a low survival rate (*p* = 0.044). Patients who died, had a positive reaction to p53. However, p53 was not observed to be a hazard factor in the event of death (HR = 39.69, *p* = 0,244).

After immunohistochemical and cytogenetic examinations, 75% of grade 2 gliomas and 25% of histopathological grade 1 gliomas were found. In our case there was a difference between the histopathological grade and age at the time of diagnosis; grade 1 gliomas developed in older people (*p* = 0.039). Furthermore, a difference was observed between grading and midline shift (*p* = 0.020). Grade 2 gliomas produced a displacement of the midline with an average of 10.08 mm, while grade 1 having 3.75 mm. This was also observed in the volumetric average of the lesions. The histopathological degree was not a predictor of mortality (HR = 2.775, *p* = 0.340).

The final diagnoses were pleomorphic xantoastrocytoma, astrocytoma NEC, oligodendroglioma, pilocytic astrocytoma, and astrocytoma IDH-mutant (Figure 5D).

There was a statistically significant difference between the different histopathological diagnoses and the displacement of the midline (*p* = 0.025). It was observed that oligodendrogliomas followed by astrocytoma IDH-mutant produced the largest displacement of the median structures. There was also a statistically significant difference between the diagnosis and gender, with a predisposition of females for pilocytic astrocytomas and astrocytoma IDH-mutant (*p* = 0.018).

**Figure 6:**
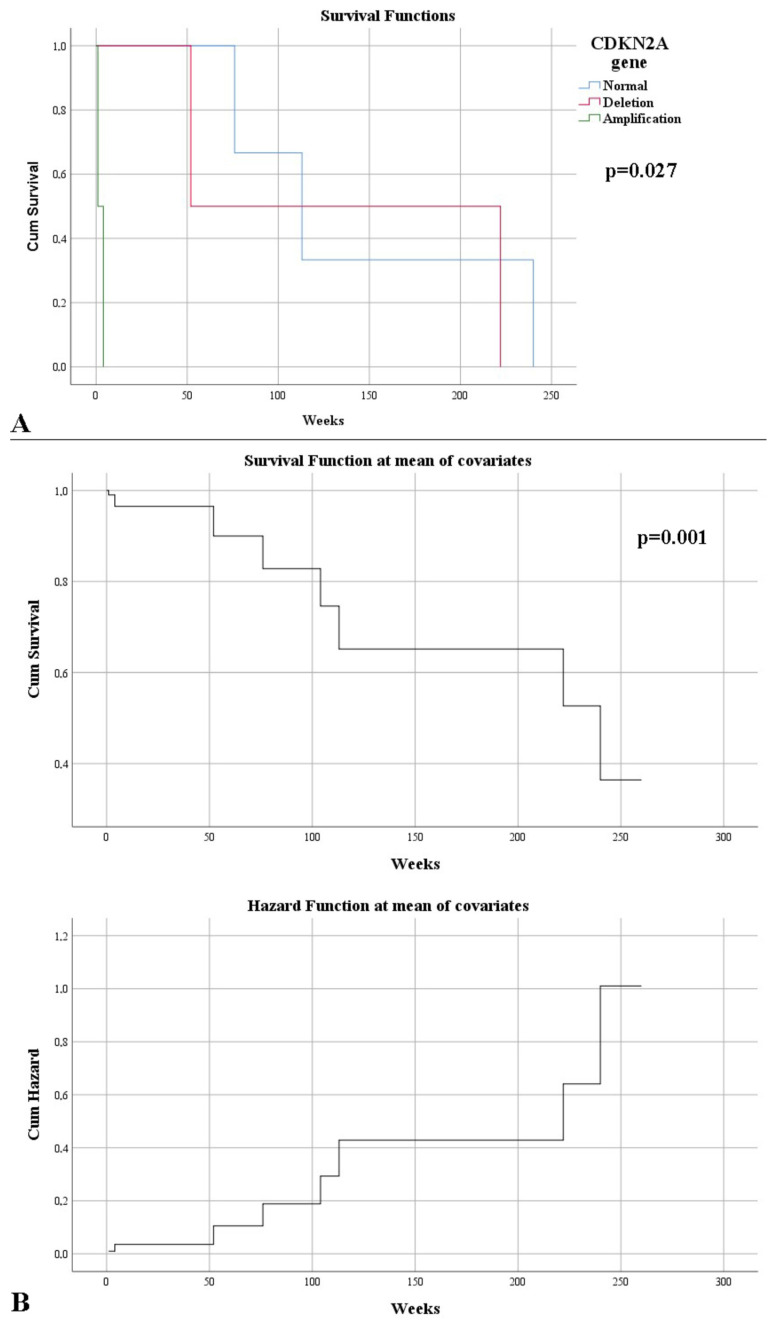
(**A**) Kaplan–Meier survival graphic that shows a lower survival rate in the case of CDKN2A mutations. (**B**) Univariate Cox regression analysis that expresses the risk of death in CDKN2A mutations.

FISH examination of the CDKN2A gene revealed mutations, such as deletion or amplification in 31.25% of cases, exclusively among grade 2 gliomas. A statistically significant correlation was observed between the type of resection, more precisely a complete resection, with a normal CDKN2A gene (*p* = 0.026). In addition, a difference was observed between the gene status and the resectability rate; thus, it was observed that a lower resectability was obtained in the case of amplifications, followed by deletions; with the normal status of the gene benefiting from an increased resectability (*p* = 0.049). An association between strong IDH1 immunointensity was correlated with the presence of CDKN2A gene mutations (*p* = 0.020). CDKN2A gene deletions and amplifications were associated with an increased proliferative index (*p* = 0.043, respectively, *p* = 0.046). At the end of the study, it was observed that those without CDKN2A abnormalities had a high survival rate (*p* = 0.009). This aspect was also indicated by the correlation between the presence of mutations and the death of patients (*p* = 0.026). Cases with normal CDKN2A had a higher survival rate (143 weeks), and where amplifications were found, the survival rate was low (*p* = 0.027) (Figure 6A). Therefore, CDKN2A was an independent risk factor for death (HR = 13.64, *p* = 0.001) (Figure 6B).

## 4. Discussion

Although a pathology not as common as other tumor entities, gliomas have a lethal evolution [9,10]. Globally, the incidence of tumors of the nervous system is about 13 per 100,000 inhabitants, and the incidence of gliomas is 6.1 per 100,000 [11]. European countries have an incidence of gliomas of about 7.3 per 100,000 inhabitants, with an increase in their incidence in the Nordic countries (about 8.82 per 100,000) [9,11,12]. Of these, low-grade gliomas of adults have a very low frequency, between 3% in the French and 7.3% in Americans [10,11]. At the European level, Romania reported an average of 10 LGG per year [13]. Our study showed that in the Dobrogea region there are about 2.67 LGG per year.

Low-grade gliomas are most often found in males (55–60%), while our study showed the opposite [4,10,14]. Differences were observed in the pathogenesis and evolution of low-grade gliomas in terms of female and male sex, with some genes having a protective role for one category and a negative prognosis for the other. However, the exact sequence of events is not known, and more studies are needed to deepen the situation [15,16,17]. As in our study, gender was not a prognostic factor for low-grade gliomas [14,18].

The most common reasons for seeing a doctor were epilepsy (37%), headache (27%), and motor deficits (21%) [19,20]. The same three symptoms were predominant in the cases we studied. Epileptic seizures were the first symptom of LGG in over 70% of cases [21]. The most common manifestations were found when the tumor lesion was in the frontal lobe [22,23,24]. This aspect was also found in our cases, the tumor localization at the level of the frontal lobe or in contact with it most frequently generated epileptic seizures. These are caused either by the mass effect of the tumor or by various factors secreted by the tumor cells, which disrupt the microenvironment adjacent to the lesion [23].

Researchers have shown that epilepsy, as the only symptom, had a positive prognosis [24,25]. However, being associated with motor or cognitive deficits determined a low survival, a feature that was also evident in our work [24]. The association of epilepsy with elevated levels of tumor proliferation has been demonstrated, in terms of increased frequency and decreased drug control [23]. In our case, similarly, patients with high Ki-67 had more frequent seizures, but without a statistically significant correlation (*p* = 0.145). In terms of survival, the presence of epilepsy had to be associated with a small tumor volume and a total resection to be a good prognostic factor [21,23]. Moreover, the presence of calcifications in the tumor was associated with a good management and prognosis of epileptic seizures [23]. Our research supports this, corroborating that epilepsy was a negative prognostic factor, along with the fact that patients diagnosed with oligodendrogliomas did not have epilepsy. Recent studies have identified that the presence of the IDH1 mutation is strongly associated with seizures. It is postulated that oncometabolite 2-hydroxyglutarate would have an excitatory effect on foci, especially in grade 2 gliomas [26,27]. IDH1 inhibitor therapies could be used for symptomatic management, in conjunction with surgery [27]. Although the resection was complete, recurrence and refraction on treatment were quite common in LGG compared to other tumors of the central nervous system [21,28].

Headache is caused by the tumor volume, which compress the adjacent parenchyma [19]. A correlation between headache and tumor volume was also found in our study. Headache, as an evocative symptom, could categorize low-grade gliomas as incidental. Survival was found to be higher in patients with headache [29]. In our study, survival was lower. This can be explained by the fact that this symptom was prevalent in females. Women presented late to the doctor, unlike males, who had a presentation in the first 3 weeks after the onset of symptoms.

Even though it is a form of onset of symptoms, motor deficits are more often associated with the end of life period [20].

The presumptive diagnosis of low-grade gliomas was realized imagistically. The main technique is magnetic resonance imaging. This also has the role of providing tumor boundaries and tumor volume [30]. Most LGGs are supratentorial, having no predilection for the hemisphere, whereas the most common location is in the frontal lobe [3,22,25].

Size is an important element in the prognosis, as well as in the risk of malignancy. There is no standard for the cut-off of axial dimensions; some authors consider a value of 5 cm, others 6 cm. In these situations, any higher value was correlated with an increased risk of mortality [24,31]. In our study the average size was below those values and did not correlate with an increased mortality. In contrast, a larger axial diameter was associated with a faster presentation to a physician for specialized treatment.

The most common LGGs had a volume below 50 cm^3^, with grade 2 occupying a larger area between 50.1 and 100 cm^3^ [32,33]. An increased volume is associated with tumor residue, but also with a low life expectancy [32,33]. In our case, tumor volume was not statistically significantly correlated with survival, but only with the appearance of symptoms.

Regarding the displacement of the midline, recent studies have identified two particular issues, with genetic correlations, regarding the prognosis and evolution of the tumor. The first aspect is represented by the midline shift in relation to the status of the IDH gene, in the cases of grade 2 glioma [34]. Thus, grade 2 gliomas with wild-type status have a higher rate of displacement of the midline, having a more aggressive character, which makes life expectancy low [34]. In our study, mutant status produced a greater displacement of the midline (9.54 mm vs. 4 mm), without a statistically significant correlation (*p* = 0.082), but it should be noted that, in our case, grade 1 gliomas were also included. In addition, the midline shows a more accentuated displacement in the case of the presence of the 1p/19q codeletion [35]. Corroborating these data, our study also observed that oligodendrogliomas produced a more pronounced displacement (12.25 mm) compared to astrocytomas IDH-mutant, grade 2 (10.81 mm) (*p* = 0.066).

The second aspect is the potential for malignant transformation of LGG. In addition to genetic criteria (Rb1 deletions, CDKN2A mutations or tp53 overexpression), a displacement of more than 10 mm was correlated with the potential for malignant evolution [36]. It can be concluded that the relationship of gliomas with midline shifts is very close. This aspect was highlighted in our research, with displacement of the midline being an independent factor in predicting mortality.

The role of imaging is obvious, even after treatment, through control examinations to monitor the presence or absence of tumor residue. In the past, complete macroscopic resection did not provide benefits in addition to a subtotal resection [37]. This aspect was contradicted by our study. In subtotal resections, even an increased resectability rate (92.45%) is insufficient to increase survival, the optimal method being complete ablation. One controversy is related to extended maximization of resection. Studies have shown that this method has been linked to increased survival in the case of wild-type LGG. Other studies showed that the technique is more suitable for the LGG IDH mutant without 1p/19q codeletion [38,39,40]. The point of convergence is that the greatest survival is achieved in the case of complete resection [37,38,39,40]. The same aspect was highlighted in our study, with complete tumorectomy, regardless of IDH status (mutant or wild-type), having a good prognosis in terms of patient survival.

Regarding pathogenesis, the most commonly involved pathway in their tumorigenesis is MAPK [3]. To this are added fusions of the BRAF kinase domain and/or alterations of the Pi3K/AKT/mTOR pathway [3,41,42]. In order to evaluate the aggressiveness, we evaluated the CDKN2A gene. It is located on chromosome 9p21 and is involved in cell proliferation and apoptosis, and is found in high-grade aggressive gliomas [43,44]. Our study noted the essential role of gene mutations in survival. Alteration of the gene, of any kind, gave grade 2 gliomas an aggressive status, with an increased morality rate. Amplifications or deletions at this level were a negative individual prognostic factor, in terms of survival. In the case of grades 3 and 4 gliomas, the presence of CDKN2A mutations represents an independent risk factor, in terms of low survival and poor prognosis, but the same was not highlighted in low-grade gliomas [45,46,47]. Our study noted the importance of this gene, in terms of the survival and prognosis of patients with low-grade gliomas, and its deletions and amplifications represented independent risk factors (HR = 13.64). In the study of Reis GF et al., gene mutations were correlated with IDH mutations, giving an increased aggressivity, especially in astrocytic gliomas [43]. In gliomas of oligodendroglial origin, these alterations were correlated both with anaplasia (oligodendroglial promotion at grade 3), but also with an increased mortality [48]. Aspects related to mortality were also identified in our study, supporting the importance of studying this gene in low-grade gliomas, and not only in high-grade ones. We observed a close correlation between the increased intensity of the IDH biomarker and CDKN2A. In addition, the correlation between the CDKN2A gene and the proliferation index also supported an increase in aggression. The study conducted by Ono Y et al., noted this aspect, in their group, there was a significant increase in the proliferative index (about 10%) compared to the values in the case of an intact gene [44]. In our case, an increased index was associated with the gene mutation.

A particular and controversial aspect is the presence of the IDH mutation in the case of pilocytic astrocytoma. This is normally absent in this grade 1 glioma. However, both in our case and in recent studies, its presence has been identified in the elderly, both supratentorial and infratentorial [42,49,50]. This information, corroborating with the data extracted by Bond KM et al., may support the presence of this mutation in particular cases of pilocytic astrocytoma in the elderly population, and may suggest an element of pathogenesis associated with age [51].

Furthermore, noteworthy was the presence of an IDH–wild-type grade 2 glioma (astrocytoma grade 2 NEC), previously named astrocytoma diffuse IDH–wild-type. Such entities have been identified in the literature and are thought to represent the initial stage of a secondary glioblastoma/glioblastoma IDH–wild-type [52]. Our study highlights its aggressiveness, with CDKN2A deletion and patient death after approximately 4 years and 3 months, but cannot support its potential for malignant transformation.

Over time, attempts have been made to establish a gradation for proliferative index, but without success. The meta-analysis performed by Johannessen AL et al. indicated a mean Ki-67 of 3.05% (0.88–7.6%) for grade 2 gliomas [53]. Grade 1 has a lower index of 1.8–3.36% [54,55,56]. In our study, grade 2 gliomas had a higher index of 3.25%, possibly due to the advanced age of the patients. Grade 1 gliomas had an average Ki-67 index of 1.5%. In both cases, quantification is not an individual predictor of aggression or mortality. According to this analysis, its impact is related either to symptoms or in association with mutations in the CDKN2A gene.

We performed a multifactorial analysis that included the main independent risk factors identified in our study (mutations or amplifications of the CDKN2A gene, midline shift, and the presence of residual tumor). We observed the following aspects: CDKN2A deletions associated with midline shift presented an HR of 1.22 (*p* = 0.015), and CDKN2A deletions associated with the presence of residual tumor had an HR of 9.41 (*p* = 0.016). This highlights two particularly important aspects. The first is represented by CDKN2A deletions, which represent both an independent mortality risk factor and one associated with midline shift and the presence of tumor residue. The second aspect refers to gene amplification, which remains an independent risk factor for mortality, denoting the existence of a mechanism that determines increased aggressiveness in these situations.

The next step in pathogenesis is changes in the TP53 gene, capable of encoding the transcriptions factors and p53 suppressor [57,58]. P53 has the role of differentiating grade 1 gliomas from the process of gliosis and its presence is rarer in gliomas of oligodendroglial origin and in high grade glioma (HGG) [57,58].

The study of Mukasa A et al. pointed out the strong association of the IDH and p53 mutations, and the study by Reis GF et al. supported this aspect and highlighted the negative impact on survival, regardless of the CDKN2A gene status [43,59]. Our study completes this information by claiming that the presence of p53 immunopositivity in grade 2 gliomas is associated with a low survival rate, but its independent action is not a significant risk factor for death. Its contribution to the low survival rate of patients is also caused by the association with midline shift, which we evaluated as an independent factor in mortality.

Two elements of major importance are represented by the degree of cell differentiation and neovascularization. Therefore, we used Nestin, which belongs to the class of intermediate filaments expressed in glial stem cells [60]. It is used as a marker of cell differentiation, the intensity of which is increased in the cases of HGGs [60,61]. Its expression is negatively correlated with IDH and gives a poor prognosis in case of increased immunointensities [62]. In our situation, the cases whose reaction was strong had a higher survival (*p* = 0.038). Even though they lost their cell differentiation, the proliferation index was low (2.4% strong reaction vs. 2.5% negative reaction). This association corroborated with the correlation Ki-67–CDKN2A mutation may be the explanation. The same aspect, of a lack of correlation with the tumor grade, was remarked on in the study of Rani SB et al. [63]. Nestin is also expressed in endothelial cells and is a marker of angiogenesis [60]. This could not be assessed due to the fact that low-grade gliomas do not show vascular proliferative activity, this feature being the prerogative of HGG [60].

A limitation of this study is represented by the small group of patients; however, given their low incidence in adults and the national situation (10 cases per year reported in Romania, of which only in the Dobrogea region we identified an average of 2.6 per year), we can say that the main negative prognostic factors that shorten the survival rate are represented by the tumor volume that determines the midline shift, the status of the main genes involved in the pathogenesis (IDH and CDKN2A), and the characteristics of the neurosurgical treatment. More studies are needed on these populations and glial categories, especially in large centers specializing in malignant pathology of the central nervous system, because, despite their rarity and low histopathological grade, LGG represents a major cause of social disability and mortality.

## 5. Conclusions

Our study succeeded in evaluating low-grade gliomas from a clinical-imaging and morpho-genetic point of view, as well as identifying the main factors involved in the patient’s prognosis. Negative prognostic factors that cause a shortening of survival by direct or indirect actions are represented by the association of headache with epilepsy, tumor volume, histopathological grade, strong immunoreaction of IDH1, and immunopositivity at p53.

The most important aspect is related to tumor aggression, which implicitly determines major risk of death. Thus, the identified independent risk factors are represented by the presence of midline shift (HR = 1.15) and the presence of tumor residue (incomplete or subtotal resections) (HR = 5.47), and the most important factor is represented by mutations (deletions or amplifications) of the CDKN2A gene (HR = 13.64).
